# Radiomic features of breast parenchyma: assessing differences between FOR PROCESSING and FOR PRESENTATION digital mammography

**DOI:** 10.1186/s13244-021-01093-4

**Published:** 2021-10-21

**Authors:** Mario Sansone, Roberta Grassi, Maria Paola Belfiore, Gianluca Gatta, Francesca Grassi, Fabio Pinto, Giorgia Viola La Casella, Roberta Fusco, Salvatore Cappabianca, Vincenza Granata, Roberto Grassi

**Affiliations:** 1 Department of Electrical Engineering and Information Technologies, University of Naples “Federico II”, Naples, Italy; 2 Department of Precision Medicine Division of Radiology, University of Campania Luigi Vanvitelli, Naples, Italy; 3 Italian Society of Medical and Interventional Radiology (SIRM), SIRM Foundation, Via della Signora 2, 20122 Milan, Italy; 4 Marcianise Hospital, Caserta, Italy; 5 Medical Oncology Division, Igea SpA, Naples, Italy; 6 Division of Radiology, “Istituto Nazionale Tumori IRCCS Fondazione Pascale - IRCCS Di Napoli”, Naples, Italy

**Keywords:** Field digital mammography, Breast cancer, Dense area, Radiomics, Textural analysis

## Abstract

**Objective:**

To assess the similarity and differences of radiomics features on full field digital mammography (FFDM) in FOR PROCESSING and FOR PRESENTATION data.

**Methods:**

165 consecutive women who underwent FFDM were included. Breasts have been segmented into “dense” and “non-dense” area using the software LIBRA. Segmentation of both FOR PROCESSING and FOR PRESENTATION images have been evaluated by Bland–Altman, Dice index and Cohen’s kappa analysis. 74 textural features were computed: 18 features of First Order (FO), 24 features of Gray Level Co-occurrence Matrix (GLCM), 16 features of Gray Level Run Length Matrix (GLRLM) and 16 features of Gray Level Size Zone Matrix (GLSZM). Paired Wilcoxon test, Spearman’s rank correlation, intraclass correlation and canonical correlation have been used. Bilateral symmetry and percent density (PD) were also evaluated.

**Results:**

Segmentation from FOR PROCESSING and FOR PRESENTATION gave very different results. Bilateral symmetry was higher when evaluated on features computed using FOR PROCESSING images. All features showed a positive Spearman’s correlation coefficient and many FOR-PROCESSING features were moderately or strongly correlated to their corresponding FOR-PRESENTATION counterpart. As regards the correlation analysis between PD and textural features from FOR-PRESENTATION a moderate correlation was obtained only for Gray Level Non Uniformity from GLRLM both on “dense” and “non dense” area; as regards correlation between PD and features from FOR-PROCESSING a moderate correlation was observed only for Maximal Correlation Coefficient from GLCM both on “dense” and “non dense” area.

**Conclusions:**

Texture features from FOR PROCESSING mammograms seem to be most suitable for assessing breast density.

## Key points

- Segmentation from FOR PROCESSING and FOR PRESENTATION gave very different results.
- Bilateral symmetry was higher when evaluated on features computed using FOR PROCESSING images.
- Texture features from FOR PROCESSING mammograms seem to be most suitable for assessing breast density.

## Introduction

Female breast cancer has now surpassed lung cancer as the leading cause of global cancer incidence in 2020, with an estimated 2.3 million new cases, representing 11.7% of all cancer cases. It is the fifth leading cause of cancer mortality worldwide, with 685,000 deaths [1].

The elevated incidence rates reflect a longstanding higher prevalence of reproductive and hormonal risk factors (early age at menarche, later age at menopause, advanced age at first birth, fewer number of kids, less breastfeeding, menopausal hormone therapy, oral contraceptives) and lifestyle risk factors (alcohol intake, excess body weight, physical inactivity), also increased detection through organized or opportunistic mammographic screening [2]. An exceptionally high prevalence of mutations in high-penetrance genes, such as BRCA1 and BRCA2, in part accounts for the high incidence in Israel and in certain European subpopulations. However, breast cancer mortality has declined over the years due to multiple factors, including more sensitive screening techniques and improved treatment regimen [3].

In the last decade there has been growing consensus regarding the role of breast parenchyma as an independent risk factor for breast cancer [4–6]: consequently, a number of approaches to breast parenchyma assessment have been proposed, among which radiomic texture feature extraction is the most spread [7–9]. Radiomics is an emerging field and has a keen interest, especially in the oncology field [10–12]: it has been shown that radiomics could be predictive of TNM grade, histological grade, response to therapy and survival in various tumors [13–15]. Textural radiomic features of breast parenchyma have been shown to be useful for cancer classification, too [16].

Radiomics features, when associated with other important information and correlated with outcomes, can provide accurate and robust evidence-based clinical-decision support systems (CDSS). The main challenge is the optimal gathering and integration of multimodal data sources in a quantitative manner capable to deliver unambiguous clinical information that accurately and robustly enable outcome prediction as a function of the necessary decisions [17–19]. The central hypothesis of radiomics is that the quantitative individual voxel-based variables are more sensitively associated with various clinical end points compared with the more qualitative radiologic, histopathologic, and clinical data more commonly used today [17–19].

Digital processing of full field digital mammography (FFDM) has enormously increased the possibility to objectively assess textural properties of breast images. Full field digital mammography can be stored as FOR PROCESSING (original or raw images) or FOR PRESENTATION (processed images, usually via proprietary, not publicly available software). Often, in routine clinical environment only FOR PRESENTATION images are available. However, although the latter emphasize certain characteristics of the image useful for masses and calcifications detection, they might not fully retain the original information contained in the FOR PROCESSING image, potentially useful for parenchyma characterization.

Previous studies [7–9] have evaluated a number of features for breast parenchyma assessment. However, a few recent changes in the field require further deeper analysis. In particular, recently, texture features have been standardized by the Image Biomarker Standardization Initiative (IBSI) [18]. It is important therefore to perform a comprehensive evaluation of differences between FOR PROCESSING and FOR PRESENTATION using the standardized features which include several additive texture features with respect to Gastounioti et al. [7]. Moreover, in Gastounioti et al. [7], texture features have been computed using a ‘lattice’ approach for characterization of the whole breast: however, the lattice has been summarized by an overall averaging: while that approach is directed towards taking approximately into account feature variability across the breast, it does not give precise information about the dense/non-dense areas of the breast. A third point is that previous studies assessed only two mammographic equipment (Siemens and Hologic) [7–9]: it is of course of interest to test whether results can be extended to other manufacturers.

The objective of our study was to assess the similarity and differences of radiomics features on FFDM in FOR PROCESSING and FOR PRESENTATION. Expanding previous studies, we addressed the problem using an enlarged set of texture radiomic features, dense/non-dense areas comparison and a new manufacturer; appropriate statistical analysis has been used.

## Methods

### Study population

Study population included 165 women who underwent mammography at the Breast Unit of the University Hospital “Luigi Vanvitelli” in Naples, Italy, from June 2020 to November 2020. The study was approved by local ethical committee and each patients enrolled have signed the informed consensus. Patients’ characteristics have been summarized in Table 1. Breast density of the sample has been assessed by two expert radiologists in consensus (G.G., M.P.B.) according to BI-RADS 5th edition published in 2013 [20]. It should be underlined that according to [20] “if the breasts are not of apparently equal density, the denser breast should be used to categorize breast density”. Therefore, only one category per each woman was available.

**Table 1:** Study population

Number of women	165
Age (mean ± SD)	56.4 y ± 9.1
Age at first menstrual period (mean ± SD)	12.0 y ± 1.6
Women in menopause	116
Age menopause (mean ± SD)	49.8 y ± 5.0
BMI (mean ± SD)	25.4 kg/mm^2^ ± 4.3
Pregnancy after 30yrs	41 (31.2%)
No childbirth	34 (20.6%)
*BIRADS density*	
Type A	14 (8.5%)
Type B	80 (48.5%)
Type C	35 (21%)
Type D	36 (22%)

BMI; *BIRADS* breast imaging reporting and data system

### Equipment and images

Women have been imaged according to current guidelines consisting of Full Filed Digital mammography (FFDM) in both mediolateral oblique (MLO) and cranio-caudal views (CC) using the system Giotto Class produced by IMS GIOTTO S.p.A. (Sasso Marconi–Bologna Italy). The specific operating conditions of mammographic image acquisition have been summarized in Table 2. Specifically, we highlight that the mammography was equipped with a tungsten anode. Tungsten anode has been shown to reduce administered dose while preserving image quality [21, 22]. For this work only MLO images have been considered because of the larger presence of breast parenchyma on this kind of projection: a total of 330 images (left/right) have been used.

**Table 2:** Equipment characteristics

Anode material	Tungsten (W)
Filter materials	0.05 mm Silver (Ag); a 0.7 mm Aluminum (Al) filter may be also available on the system
Detector	a-Se flat panel detector
Pixel size	85 µm
kVp (median, range)	31 (26–35)
Exposure time (ms) (median, range)	516 (285–1340)
mAs (median, range)	77 (39–200)
Anode/filter combination	W / Ag
Entrance dose (mGy)	5.01 (1.51–15.1)

### Breast segmentation

Breasts have been segmented into “dense” area (roughly corresponding to the fibroglandular tissue) and “non-dense” area (the remaining part of the breast) using the publicly available softare LIBRA [8, 9] available for MATLAB (Version: 9.3.0.713579, R2017b. Natick, Massachusetts: The MathWorks Inc.). LIBRA has been specifically developed for breast segmentation, pectoral muscle removal and percent density computation. Both FOR PROCESSING and FOR PRESENTATION images from our dataset have been tested for proper segmentation. Bland–Altman, Dice index and Cohen’s kappa analysis (“Statistical analysis” section) has been used to assess differences between the two types of segmentation. Subsequently, radiomic features have been computed both on “dense” and “non-dense” area and on FOR PROCESSING and FOR PRESENTATION images. Percent density from LIBRA has also been computed.

Before LIBRA segmentation, FOR-PROCESSING images underwent minimal pre-processing: logarithm and *z*-scoring; FOR-PRESENTATION images were subjected only to *z*-score to align image histogram to FOR-PROCESSING image [7, 9].

It should be emphasized that LIBRA has been developed on equipment by two specific manufacturers (Siemens and Hologic). One of the objective of our analysis was to assess whether LIBRA could be used reliably on a different manufacturer (IMS GIOTTO S.p.A.) without any modification.

### Radiomic features

Recently, the IBSI [18] has standardized a set of 174 features. Such features have been implemented in PyRadiomics [19] a library available within Python environment [23]. Briefly, IBSI features include texture and morphological features. In this study we considered only textural features. In fact, it has been suggested in literature that texture feature might well describe parenchymal structure [7, 8, 20].

**Fig. 1:**
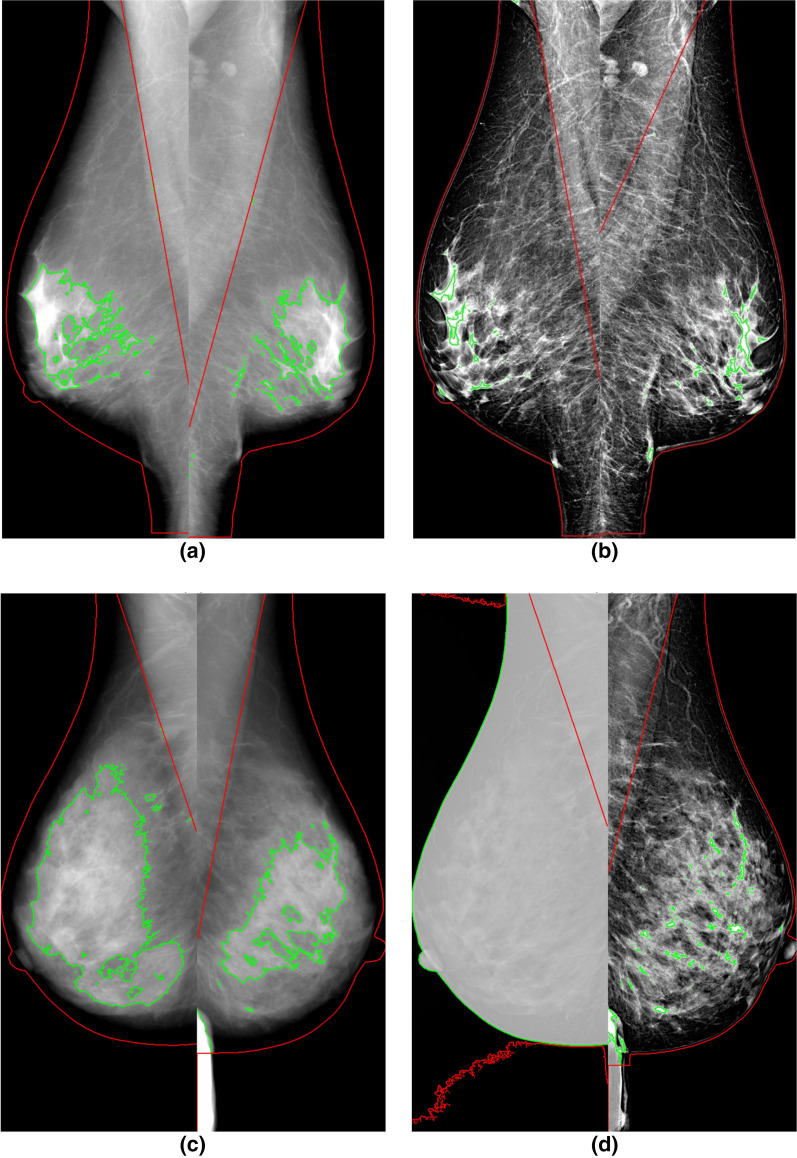
Representative examples of segmentation of FFDM using LIBRA. **a** Segmentation using FOR PROCESSING images; **b** the same breasts segmented using FOR PRESENTATION images. In red the breast area; in green the dense area. There are large differences in the main breast area (pectoral muscle removal) and in the dense area. **c**, **d** An example from another woman representative of very large errors with consequently wrong Percent Density (PD)

Seventy-four textural features were used in this study, grouped into 4 main groups: 18 features of First Order (FO), 24 features of Gray Level Co-occurrence Matrix (GLCM), 16 features of Gray Level Run Length Matrix (GLRLM) and 16 features of Gray Level Size Zone Matrix (GLSZM). See Table 3 for a list of all features. A detailed description of each textural feature is reported in the website https://pyradiomics.readthedocs.io/en/latest/features.html. Features have been computed both on dense and non-dense breast areas (see Fig. 1).

**Table 3:** Comparison between FOR-PROCESSING and FOR-PRESENTATION features computed on “dense” area and “non-dense” area

Feat group	Feature name	*w*	*r* (dense)	*r* (no dense)	Can Cor	PD FOR PRES dense	PD FOR PROC dense	PD FOR PRES no-dense	PD FOR PROC no-dense
1st or	10 percentile		0.99	1.00	1.00	− 0.21	− 0.16	− 0.15	− 0.17
	90 percentile		0.99	1.00	1.00	− 0.21	− 0.11	− 0.16	− 0.15
	Energy		1.00	1.00	1.00	0.51	0.54	− 0.45	− 0.45
	Entropy		0.57	0.64	0.71	0.13	0.55	0.25	0.39
	Interquartile Range		0.50	0.30	0.50	0.17	0.71	− 0.24	0.36
	Kurtosis		0.57	0.92	0.92	− 0.37	− 0.62	(a)	− 0.15
	Maximum		0.78	0.57	0.84	− 0.20	− 0.12	− 0.35	− 0.15
	Mean Absolute Deviation		0.47	0.61	0.76	(a)	0.66	− 0.15	0.13
	Mean		0.99	1.00	1.00	− 0.21	− 0.13	− 0.17	− 0.17
	Median		1.00	1.00	1.00	− 0.20	− 0.13	− 0.17	− 0.18
	Minimum		0.97	–	0.98	− 0.24	− 0.16	–	–
	Range		0.59	0.57	0.80	(a)	0.42	− 0.35	− 0.15
	Robust Mean Absolute Deviation		0.49	0.29	0.52	0.15	0.71	− 0.26	0.28
	Root mean squared		0.99	1.00	1.00	− 0.21	− 0.13	− 0.16	− 0.17
	Skewness	(a)	0.64	0.92	0.92	− 0.49	− 0.58	(a)	0.13
	Total energy		1.00	1.00	1.00	0.51	0.54	− 0.45	− 0.45
	Uniformity		0.55	0.61	0.74	− 0.16	− 0.59	− 0.23	− 0.45
	Variance		0.44	0.94	0.99	(a)	0.60	(a)	0.08
glcm	Autocorrelation		0.51	0.40	0.55	0.31	0.36	0.21	− 0.31
	Cluster Prominence		0.39	0.71	0.53	− 0.16	0.35	0.18	0.06
	Cluster Shade		0.40	0.57	0.50	− 0.37	(a)	− 0.15	− 0.06
	Cluster tendency		0.50	0.68	0.70	(a)	0.48	0.21	0.14
	Contrast		0.69	0.69	0.80	− 0.15	− 0.44	0.39	0.32
	Correlation		0.63	0.77	0.73	0.34	0.66	− 0.35	− 0.26
	Difference average		0.71	0.73	0.77	− 0.11	− 0.39	0.37	0.48
	Difference entropy		0.71	0.75	0.79	− 0.12	− 0.39	0.35	0.50
	Difference variance		0.68	0.76	0.85	− 0.21	− 0.51	0.40	0.30
	Inverse Difference (ID)	(a)	0.72	0.72	0.78	(a)	0.36	− 0.32	− 0.47
	Inverse Difference Moment (IDM)	(a)	0.72	0.72	0.78	(a)	0.36	− 0.31	− 0.47
	Inverse Difference Moment Normalized (IDMN)		0.70	0.70	0.79	0.14	0.43	− 0.39	− 0.33
	Inverse Difference Normalized (IDN)		0.71	0.74	0.77	(a)	0.38	− 0.36	− 0.49
	Informational Measure of Correlation (IMC) 1		0.66	0.67	0.70	− 0.30	− 0.65	0.38	0.48
	Informational Measure of Correlation (IMC) 2		0.65	0.70	0.68	0.32	0.67	− 0.31	− 0.12
	Inverse Variance	(a)	0.72	(a)	0.78	(a)	0.35	− 0.31	− 0.10
	Joint Average		0.47	0.42	0.58	0.32	0.29	0.21	− 0.29
	Joint Energy		0.66	0.64	0.75	(a)	− 0.28	− 0.28	− 0.49
	Joint Entropy		0.69	0.68	0.79	(a)	0.17	0.30	0.46
	Maximal Correlation Coefficient (MCC)		0.48	0.73	0.75	(a)	0.60	− 0.43	− 0.61
	Maximum Probability	(a)	0.60	0.61	0.75	− 0.12	− 0.36	− 0.30	− 0.49
	Sum Average		0.47	0.42	0.58	0.32	0.29	0.21	− 0.29
	Sum entropy		0.59	0.64	0.71	0.15	0.54	0.24	0.40
	Sum squares		0.49	0.66	0.66	(a)	0.44	0.25	0.16
glszm	Gray Level Non Uniformity		0.88	0.89	0.92	0.72	0.50	− 0.56	− 0.62
	Gray Level Non Uniformity Normalized		0.54	0.65	0.72	− 0.15	− 0.59	− 0.29	− 0.52
	Gray Level Variance		0.46	0.64	0.72	(a)	0.46	0.39	0.43
	High Gray Level Zone Emphasis		0.52	0.29	0.49	0.30	0.40	0.21	− 0.43
	Large Area Emphasis		0.74	0.22	0.44	0.16	0.40	− 0.18	− 0.51
	Large Area High Gray Level Emphasis		0.71	0.74	0.60	0.56	0.60	− 0.17	− 0.50
	Large Area Low Gray Level Emphasis		0.43	0.88	0.96	− 0.15	0.16	(a)	− 0.17
	Low Gray Level Zone Emphasis		0.25	0.72	0.65	− 0.35	(a)	− 0.13	− 0.04
	Size Zone Non Uniformity		0.89	0.74	0.90	0.64	0.71	− 0.29	− 0.41
	Size Zone Non Uniformity Normalized		0.75	− 0.38	0.78	− 0.18	− 0.39	0.17	− 0.49
	Small Area Emphasis		0.75	− 0.37	0.78	− 0.18	− 0.39	0.17	− 0.49
	Small Area High Gray Level Emphasis		0.52	0.18	0.50	0.25	0.33	0.22	− 0.47
	Small Area Low Gray Level Emphasis		0.22	0.40	0.29	− 0.40	− 0.15	− 0.21	− 0.29
	Zone Entropy	(a)	0.73	0.28	0.75	0.40	0.66	0.12	0.52
	Zone Percentage		0.75	0.68	0.79	− 0.16	− 0.39	0.22	0.24
	Zone Variance		0.74	0.20	0.43	0.15	0.40	− 0.17	− 0.51
glrlm	Gray Level Non Uniformity		0.89	0.88	0.89	0.66	0.50	− 0.61	− 0.62
	Gray Level Non Uniformity Normalized		0.55	0.62	0.74	− 0.16	− 0.59	− 0.24	− 0.49
	Gray Level Variance		0.49	0.53	0.66	(a)	0.47	0.36	0.28
	High Gray Level Run Emphasis		0.52	0.37	0.52	0.31	0.39	0.21	− 0.36
	Long Run Emphasis		0.74	0.63	0.75	0.15	0.39	− 0.26	− 0.33
	Long Run High Gray Level Emphasis	(a)	0.50	0.68	0.84	0.36	0.47	(a)	− 0.32
	Long Run Low Gray Level Emphasis	(a)	0.29	0.98	0.96	− 0.29	(a)	(a)	− 0.02
	Low Gray Level Run Emphasis		0.24	0.78	0.75	− 0.34	(a)	0.43	0.33
	Run Entropy		0.60	0.66	0.71	0.23	0.61	0.20	0.20
	Run Length Non Uniformity		0.98	0.71	0.98	0.79	0.80	− 0.47	− 0.24
	Run Length Non Uniformity Normalized		0.75	0.68	0.80	− 0.16	− 0.39	0.21	0.26
	Run Percentage		0.75	0.69	0.79	− 0.16	− 0.39	0.21	0.31
	Run Variance		0.74	0.55	0.73	0.15	0.39	− 0.26	− 0.34
	Short Run Emphasis		0.75	0.68	0.79	− 0.16	− 0.39	0.21	0.24
	Short Run High Gray Level Emphasis		0.52	0.18	0.48	0.29	0.37	0.23	− 0.02
	Short Run Low Gray Level Emphasis		0.23	0.70	0.62	− 0.36	(a)	0.28	0.16

The columns have the following meanings: *w*: results of paired Wilcoxon test between FOR-PROCESSING and FOR PRESENTATION: as regads dense-area non-significant (*p* > 0.05) test have been marked with “(a)”; as regads no-dense area all features were significantly different; *r*: Spearman’s correlation coefficient (they are all statistically significant, *p* < 0.05); *CanCor*: canonical correlation (see text for details); *PD-FOR-PRES and PD-FOR-PROC*: Spearman’s correlation coefficient between PD and features computed on FOR-PRES and FOR-PROC respectively, non-significant correlation (*p* > 0.05) have been marked with (a). For feature named ‘Minimum’ it was not possible to compute Spearman correlation on no-dense area because the value is always 0

### Statistical analysis

Our analysis had the objective to assess differences between features computed from FOR PROCESSING and from FOR PRESENTATION images on both ‘dense’ and ‘non-dense’ areas of the breast.

First, we assessed differences in LIBRA breast area (dense or non-dense) segmentation using Bland–Altman, Dice index and Cohen’s kappa analysis [24]: while the first is mainly a graphical approach and has been performed on the area expressed in cm^2^, the other two give an agreement measure (Dice is between 0 and 1, while kappa is with − 1, 1) between the two segmentations. Bilateral symmetry (correspondence in breast area and percent density between left/right breast) was also used to evaluate goodness of segmentation. The objective of this analysis was to verify that LIBRA processing was sufficiently accurate for the equipment from IMS GIOTTO S.p.A., as this equipment has not been tested previously for LIBRA.

Second, for each feature, Wilcoxon paired test has been applied between FOR-PROCESSING versus FO-PRESENTATION. As a further measurement, Spearman’s rank correlation coefficient has been evaluated. Canonical correlation analysis has been used to assess the correlation of linear combination of dense/non-dense features between FOR PROCESSING and FOR PRESENTATION [25].

Third, percent density (PD) correlation with each feature has been assessed via Spearman’s coefficient. Finally, for each feature bilateral symmetry (correspondence between the two breasts of the same woman) has been assessed using intraclass correlation coefficient (ICC) [7].

Dependence of correlation from equipment and women factors such as kVp, mAs, body part thickness, body mass index (BMI), age, menopause has been assessed via linear mixed effect models [26].

## Results

### Segmentation assessment

**Fig. 2:**
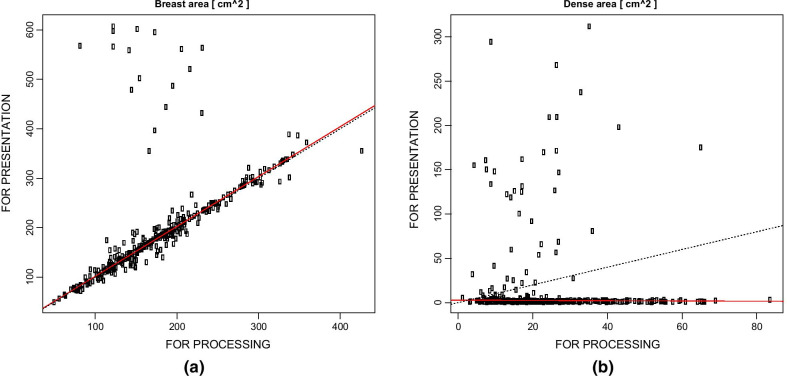
Bland–Altman analysis of (**a**) breast and (**b**) dense area (cm^2^) segmented with LIBRA on FOR PROCESSING and on FOR PRESENTATION images. Each marker represents an image. Robust linear fitting is reported in red (solid); identity line is dotted for reference. Spearman’s rank correlation index is (**a**) 0.88, (**b**) − 0.16 (*p* < 0.001). While a strong correlation exists betweeen breast areas, the negative correlation coefficient for dense area can be understood by the example in Fig. 1: often the dense area segmented on FOR PRESENTATION images is very small compared to the dense area segmented from FOR PROCESSING

In Fig. 1 we reported an exemplificative case of breast area (whole breast without pectoral muscle, dense area, non-dense area) segmentation: FOR PROCESSING and FOR PRESENTATION images gave very different results. This can be further appreciated in Fig. 2a, b reporting the Bland–Altman analysis of the whole breast and dense area. Dice index and Cohen’s kappa applied to the whole breast gave an average agreement of 0.97 ± 0.02 and 0.96 ± 0.03 respectively.

**Fig. 3:**
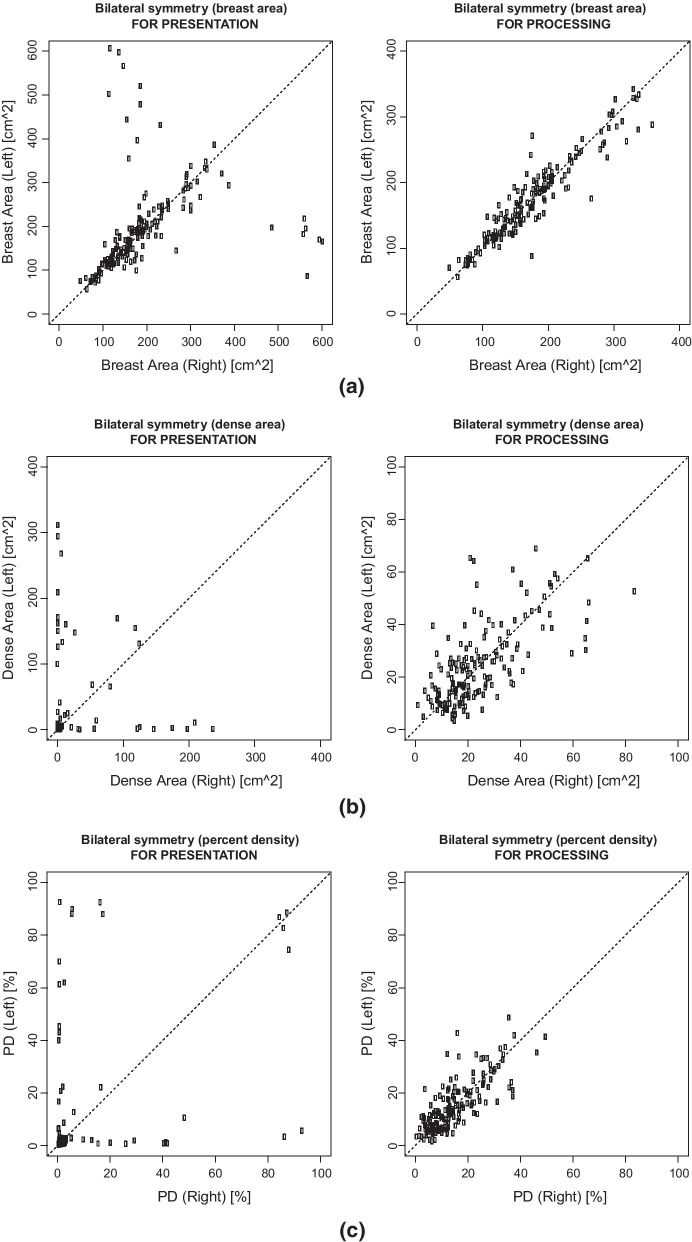
Bilateral simmetry with respect to breast-area (**a**), dense-area (**b**) and percent-density (**c**). Each circle is a woman. Women with strong bilateral simmetry should be aligned with the identity line (dashed)

As regards dense and non-dense area, as can be seen in Figs. 1 and 2a, often dense area segmented on FOR PROCESSING was very small with respect to the FOR PROCESSING counterpart. In this case it was not possible to use Dice index or Cohen’s kappa and bilateral symmetry for breast and dense areas have been evaluated: results have been reported in Fig. 3 showing that bilateral symmetry was higher when using FOR PROCESSING images.

Recognizing these limitations and such large differences between breast areas, for subsequent feature computation we decided to use only the segmentation of dense and non-dense areas from FOR PROCESSING images.

### Features assessment

Table 3 reports association between features computed on FOR-PROCESSING and FOR-PRESENTATION images over segmented dense and non-dense areas: Spearman’s correlation coefficients rho and *p* value of Wilcoxon test have been reported (significance level *p* = 0.05). Almost all texture features were significantly different, only 8 features (indicated with ‘a’ in the table) were not different according to Wilcoxon test: Skewness of FO; Inverse Difference (ID), Inverse Difference Moment (IDM), Inverse Variance, Maximum Probability of GLCM and Small Area Low Gray Level Emphasis, Long Run High Gray Level Emphasis, Long Run Low Gray Level Emphasis of GLSZM.

However, all Spearman’s correlations were positive: in particular, 12 features had a strong correlation (rho ≥ 0.8), 30 had moderate correlation (rho ≥ 0.6), 25 were weakly correlated (rho ≥ 0.4), 7 were practically uncorrelated (rho < 0.4); by visual inspection, for strongly and moderately correlated features the relationship was approximately linear.

Considering canonical correlation (Table 3, Can Cor column) to asses association between FOR-PROCESSING and FOR-PRESENTATION of combination of dense plus non dense area, all features had a moderate correlation (Can Cor ≥ 0.6) except that Interquartile Range and Robust Mean Absolute Deviation of FO, Autocorrelation, Cluster Prominence, Cluster Shade, Joint Average and Sum Average of GLCM and High Gray Level Zone Emphasis, Large Area Emphasis, Small Area High Gray Level Emphasis, Small Area Low Gray Level Emphasis, Zone Variance, High Gray Level Run Emphasis, Short Run High Gray Level Emphasis of GLSZM. Among these only Small Area Low Gray Level Emphasis was included among the 8 features not different according to Wilcoxon test between FOR-PROCESSING and FOR-PRESENTATION features.

Considering the correlation analysis between PD and textural features:

- using FOR-PRESENTATION data on “dense” area (Table 3, PD FOR PRES dense column), a moderate correlation was obtained for Gray Level Non Uniformity of GLSZM and Size Zone Non Uniformity, Gray Level Non Uniformity and Run Length Non Uniformity of GLRLM;
- using FOR-PRESENTATION data on “non-dense” area (Table 3, PD FOR PRES non-dense column), a moderate correlation was obtained only for Gray Level Non Uniformity of GLRLM (this feature was included among the 4 features with moderate correlation on “dense” area);
- using FOR-PROCESSING data on “dense” area (Table 3, PD FOR PROC dense column), a moderate correlation was obtained for Interquartile Range, Kurtosis, Mean Absolute Deviation, Robust Mean Absolute Deviation, Variance of FO; correlation; Informational Measure of Correlation (IMC) 1, IMC2, Maximal Correlation Coefficient (MCC) of GLCM and Large Area High Gray Level Emphasis, Size Zone Non Uniformity, Zone Entropy, Run Entropy, Run Length Non Uniformity of GLSZM;
- using FOR-PROCESSING data on “non-dense” area (Table 3, PD FOR PROC no-dense column), a moderate correlation was obtained for MCC of GLCM and Gray Level Non Uniformity obtained of GLSZM and of GLRLM (only MCC of GLCM was included among the 14 features with moderate correlation on “dense” area).

### Other findings

**Fig. 4:**
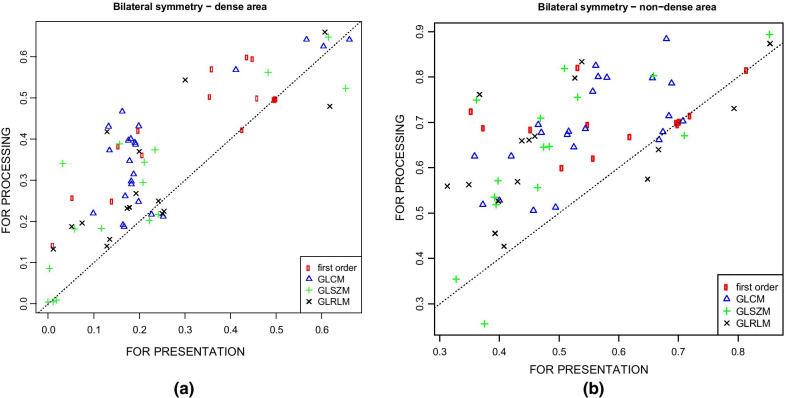
Feature bilateral symmetry on dense area (**a**) and on non-dense area (**b**). Per each feature, computed both on FOR PROCESSING and on FOR PRESENTATION, ICC between left and right breast has been evaluated. Each marker in the plot represents a feature; dotted identity line is reported as a reference. Markers appear to lie mainly above the identiy line suggesting bilateral symmetry is higher when evaluated on features computed using FOR PROCESSING images

In Fig. 4 was reported the bilateral symmetry (intra-class correlation coefficient between left/right breast) per each feature on dense and non-dense areas. It can be seen that bilateral symmetry is higher when feature are computed on FOR PROCESSING images.

**Fig. 5:**
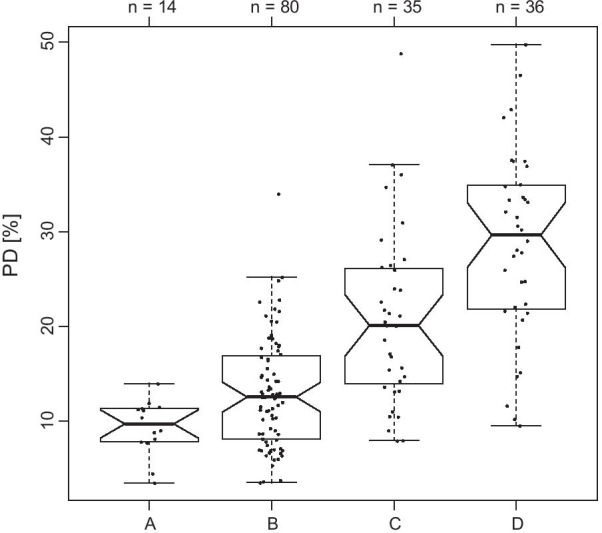
Percent density association with BI-RADS assigned by radiologists. Each point is a woman

In Fig. 5, percent density (PD) association with BI-RADS assigned by radiologists was reported. Kruskal–Wallis test was significant (*p* < 0.001). Multiple comparison test (Tukey HSD) indicates that BI-RADS density A is not significantly different from BI-RADS density B (*p* > 0.05).

**Fig. 6:**
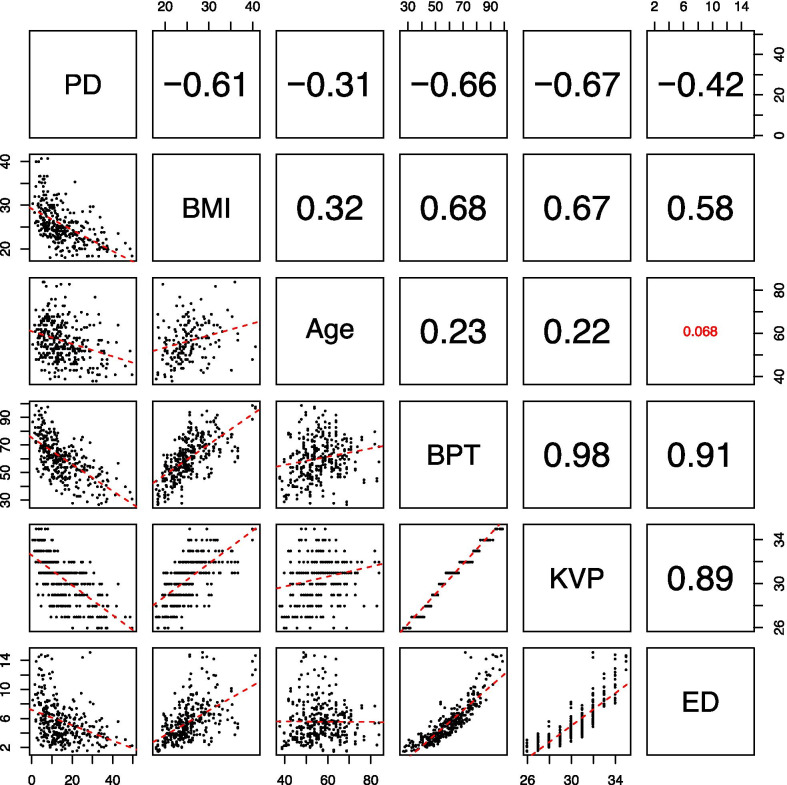
Correlations among equipment variables and patient features: per each couple of variables, scatter plot and spearman correlation value have been reported. Lower matrix: scatter plot; Upper matrix: correlation with Spearman Correlation Coefficient.The Spearman correlation coefficient, rs, can take values from + 1 to − 1. A *rs* of + 1 indicates a perfect association of ranks, a *rs* of zero indicates no association between ranks and a *rs* of − 1 indicates a perfect negative association of ranks. The closer *rs* is to zero, the weaker the association between the ranks. *PD* percent density (%), BMI; *BPT* body part thickness (mm), *KVP* kVp; *ED* entrance dose (mGy). In small font (red in color version) not significant correlations (*p* > 0.05)

No significant dependence of the correlation from equipment and women factors such as kVp, mAs, part thickness, BMI, age, menopause assessed via linear mixed effect models was found. Weak correlations were observed between equipment variables (PD, BMI, Age) and patient features (BPT, KVP, ED) (Fig. 6).

## Discussion

In the last two decades FFDM has replaced screen film mammography (SFM) in breast cancer screening [27–29]. FFDM image acquisition initially generates an image which is proportional to the X-ray attenuation through the breast, known as the raw image (i.e., FOR PROCESSING; often with a 14-bit gray-level depth). Then, vendor specific post-processing algorithms are applied to increase lesion conspicuity before radiological presentation, creating what is known as the processed image (i.e., FOR PRESENTATION; often with a 12-bit gray-level depth). It seems reasonable to assume that breast parenchyma analysis should be performed directly from raw images since they retain the original relationship with the physical properties of the breast tissue [5–9].

In this study we assessed differences between texture features computed on automatically segmented dense (manly fibro-glandular) and non-dense (mainly fat) area within the breast both on FOR PROCESSING and on FOR PRESENTATION data.

Our findings can be resumed as follows. Mainly, all features showed a positive Spearman’s correlation coefficient and many feature of FOR-PROCESSING were moderately or strongly correlated to their corresponding FOR-PRESENTATION counterpart; nonetheless, Wilcoxon test suggested differences for most of the features except for ID, IDM, Inverse Variance, Maximum Probability of GLCM and Small Area Low Gray Level Emphasis, Long Run High Gray Level Emphasis, Long Run Low Gray Level Emphasis of GLSZM.

Moreover, our results showed that the segmentation from FOR PROCESSING and FOR PRESENTATION might give very different results: the breast area segmented from the FOR PRESENTATION images is different because the pectoral muscle has not been properly removed. Moreover, often the dense area is really very small when segmented on FOR PRESENTATION: this might cause loss of potentially important texture information. In addition, the bilateral symmetry was higher when using features computed form FOR PROCESSING images.

As regards, the correlation analysis between PD and textural features on FOR-PRESENTATION a moderate correlation was obtained only for Gray Level Non Uniformity of GLRLM both on “dense” and “non dense” area. On the other side, considering the correlation analysis between PD and textural features on FOR-PROCESSING a moderate correlation was observed only for MCC of GLCM both on “dense” and “non dense” area.

Our results are in line with the findings in [7]; however a number of differences with that study must be highlighted. First, in [7] a limited number of features (28) has been investigated; however, thanks to the effort of IBSI [18] it is today possible to examine a very large number of features. In our study we used 74 features (computed on the original image without wavelet transform) subdivided into four main groups. Moreover, the group of GLSZM has not been investigated at all in [7].

Second, in [7] a “lattice” approach has been used to compute features, however an averaging over the lattice has been made to resume the behavior of the breast; in our study, instead, we segmented the breast into two main regions “dense” and “non-dense” and the correlation between features have been searched in both regions separately and concurrently (canonical correlation analysis).

Third, a few indices used in [7] might be inappropriate for evaluating correlation: specifically, Bland–Altman analysis of breast area might miss true correspondence between areas; to this aim, we used Dice index and Cohen’s kappa for comparing non-dense areas; however, the comparison between dense areas seemed inappropriate because they were strongly different by visual inspection.

There are a number of limitations to the presented study. First, the limited size of the sample. Second, radiologist-provided estimates of breast percent density were not available for independent validation. Third, only digital mammograms from a single manufacturer (IMS Giotto S.p.a) have been analyzed.

In conclusion, segmentation results suggest that LIBRA is capable to properly segment FOR PROCESSING images from the vendor considered. As regards radiomic texture features, our results indicates that, although some features seem to be robust with respect to the type image used for computation, FOR PROCESSING mammograms may be most suitable for assessing breast density, as these images are less influenced by vendor-specific post-processing algorithms.

## Data Availability

All data are reported in the manuscript.

## Abbreviations

BMI: Body mass index
CC: Cranio-caudal
CDSS: Clinical-decision support systems
FFDM: Full field digital mammography
FO: First Order
GLCM: Gray Level Co-occurrence Matrix
GLRLM: Gray Level Run Length Matrix
GLSZM: Gray Level Size Zone Matrix
IBSI: Image Biomarker Standardization Initiative
ICC: Intraclass correlation coefficient
ID: Inverse
ID: Inverse Difference
IDM: Inverse Difference Moment
IMC: Informational Measure of Correlation
MCC: Maximal Correlation Coefficient
MLO: Mediolateral oblique
PD: Percent density
SFM: Screen film mammography

## References

[1] Sung H, Ferlay J, Siegel RL (2021). Global cancer statistics 2020: GLOBOCAN estimates of incidence and mortality worldwide for 36 cancers in 185 countries. CA Cancer J Clin.

[2] Deandrea S, Cavazzana L, Principi N (2021). Screening of women with aesthetic prostheses in dedicated sessions of a population-based breast cancer screening programme. Radiol Med.

[3] Pediconi F, Galati F, Bernardi D (2020). Breast imaging and cancer diagnosis during the COVID-19 pandemic: recommendations from the Italian College of Breast Radiologists by SIRM. Radiol Med.

[4] Pinker K (2019). Beyond breast density: radiomic phenotypes enhance assessment of breast cancer risk. Radiology.

[5] Kontos D, Winham SJ, Oustimov A (2019). Radiomic phenotypes of mammographic parenchymal complexity: toward augmenting breast density in breast cancer risk assessment. Radiology.

[6] Gastounioti A, Conant EF, Kontos D (2016). Beyond breast density: a review on the advancing role of parenchymal texture analysis in breast cancer risk assessment. Breast Cancer Res.

[7] Gastounioti A, Oustimov A, Keller BM (2016). Breast parenchymal patterns in processed versus raw digital mammograms: a large population study toward assessing differences in quantitative measures across image representations. Med Phys.

[8] Keller BM, Chen J, Daye D, Conant EF, Kontos D (2015). Preliminary evaluation of the publicly available Laboratory for Breast Radiodensity Assessment (LIBRA) software tool: comparison of fully automated area and volumetric density measures in a case-control study with digital mammography. Breast Cancer Res.

[9] Keller BM, Nathan DL, Wang Y (2012). Estimation of breast percent density in raw and processed full field digital mammography images via adaptive fuzzy c-means clustering and support vector machine segmentation. Med Phys.

[10] Granata V, Fusco R, Barretta ML (2021). Radiomics in hepatic metastasis by colorectal cancer. Infect Agent Cancer.

[11] Fusco R, Piccirillo A, Sansone M (2021). Radiomics and artificial intelligence analysis with textural metrics extracted by contrast-enhanced mammography in the breast lesions classification. Diagnostics (Basel).

[12] Granata V, Fusco R, Avallone A (2021). Radiomics-derived data by contrast enhanced magnetic resonance in ras mutations detection in colorectal liver metastases. Cancers (Basel).

[13] Danti G, Berti V, Abenavoli E (2020). Diagnostic imaging of typical lung carcinoids: relationship between MDCT, (111) In-Octreoscan and (18)F-FDG-PET imaging features with Ki-67 index. Radiol Med.

[14] Hu HT, Shan QY, Chen SL (2020). CT-based radiomics for preoperative prediction of early recurrent hepatocellular carcinoma: technical reproducibility of acquisition and scanners. Radiol Med.

[15] Farchione A, Larici AR, Masciocchi C (2020). Exploring technical issues in personalized medicine: NSCLC survival prediction by quantitative image analysis-usefulness of density correction of volumetric CT data. Radiol Med.

[16] Li H, Mendel KR, Lan L, Sheth D, Giger ML (2019). Digital mammography in breast cancer: additive value of radiomics of breast parenchyma. Radiology.

[17] Mazo C, Kearns C, Mooney C, Gallagher WM (2020). Clinical decision support systems in breast cancer: a systematic review. Cancers (Basel).

[18] Zwanenburg A, Vallières M, Abdalah MA (2020). The Image biomarker standardization initiative: standardized quantitative radiomics for high-throughput image-based phenotyping. Radiology.

[19] van Griethuysen JJM, Fedorov A, Parmar C (2017). Computational radiomics system to decode the radiographic phenotype. Cancer Res.

[20] American College of Radiology (2013). ACR BI-RADS Atlas—mammography.

[21] Oduko JM, Young KC, Gundogdu O, Alsager A, Krupinski EA (2008). Effect of using tungsten-anode X-ray tubes on dose and image quality in full-field digital mammography. Digital mammography. IWDM 2008. Lecture notes in computer science 2008.

[22] Borg M (2019). Application of the European protocol in the evaluation of digital mammography units with tungsten target tubes. Radiat Prot Dosimetry.

[23] Van Rossum G, Drake FL (2009). Python 3 reference manual.

[24] Taha AA, Hanbury A (2015). Metrics for evaluating 3D medical image segmentation: analysis, selection, and tool. BMC Med Imaging.

[25] Härdle WK, Simar L (2015). Applied multivariate statistical analysis.

[26] Tuerlinckx F, Rijmen F, Verbeke G, De Boeck P (2006). Statistical inference in generalized linear mixed models: a review. Br J Math Stat Psychol.

[27] Kallenberg MG, Lokate M, van Gils CH, Karssemeijer N (2011). Automatic breast density segmentation: an integration of different approaches. Phys Med Biol.

[28] Tagliafico A, Tagliafico G, Tosto S (2009). Mammographic density estimation: comparison among BI-RADS categories, a semi-automated software and a fully automated one. Breast.

[29] Glide-Hurst CK, Duric N, Littrup P (2007). A new method for quantitative analysis of mammographic density. Med Phys.

